# Abnormality in field physical test predicts a reduced quadriceps strength in patients with hip- or knee-osteoarthritis. A prospective observational study

**DOI:** 10.1371/journal.pone.0314524

**Published:** 2024-12-27

**Authors:** Quentin Fanget, Anargyros Verdilos, Samuel Adelou, Vivien Reynaud, Stéphane Boisgard, Stéphane Descamps, Bruno Pereira, Ruddy Richard, Emmanuel Coudeyre, Frédéric Costes

**Affiliations:** 1 CHU Clermont Ferrand, Plateforme d’Exploration de la Mobilité, Pôle MOBEX, Université Clermont Auvergne, Clermont-Ferrand, France; 2 Service de Médecine Physique et Réadaptation, Pôle MOBEX, UNH, CHU Clermont Ferrand, Université Clermont Auvergne, Clermont-Ferrand, France; 3 Direction de la Recherche Clinique et de l’Innovation, CHU Clermont Ferrand, Clermont-Ferrand, France; 4 Service d’Orthopédie, CHU Clermont Ferrand, Pôle MOBEX, Université Clermont Auvergne, Clermont-Ferrand, France; 5 INRAE, Unité de Nutrition Humaine, Université Clermont Auvergne, Clermont-Ferrand, France; Universiti Sains Malaysia, MALAYSIA

## Abstract

**Background:**

In osteoarthritis quadriceps strength is an important outcome to assess exercise capacity and recovery after arthroplasty. However, its measurement is limited due to lack of time and the need for trained personnel and equipment whose accuracy is verified.

**Objectives:**

To find out the determinants of a reduced quadriceps strength and to establish a score to screen for it.

**Methods:**

In an observational prospective study, we evaluated patients presenting with an unilateral knee (KOA) or a hip (HOA) osteoarthritis before a scheduled arthroplasty. We measured body composition, exercise capacity, muscle strength, balance, WOMAC score, quality of life and physical activity. Isometric maximal voluntary quadriceps force (MVCq) was determined on both lower limbs and a reduced strength was retained when at least one measurement was lower than 1 standard deviation of normal value.

**Results:**

We included 376 patients, 247 (66%) with KOA and 129 (34%) with HOA. Their mean age was 67±8 years, and mean BMI 31.4±6.9 kg/m^2^. MVCq was reduced in 217 (58%). Compared those with a preserved MVCq, these patients had a significant higher BMI and lean mass, a sex ratio (more men), an altered field physical tests and WOMAC score. The best logistic regression model for a decreased quadriceps force included pathology, age, sex, BMI, five sit to stand test (FTSST) and maximal gait speed (AUC was 0.87 [95%CI 0.83–0.90]). We developed a predictive equation for a reduced MVCq as follows: Y = 1/1+ exp[-(-0.051*age -1.25*max gait speed + 0.09*FTSST + 0.16*BMI + 1.1 (for KOA) + 2.41 (for male) -1.79].

**Conclusion:**

MVCq is reduced with a high prevalence on patients with KOA or HOA. A low performance in one the selected field physical test associated with age and BMI allows to screen for those in whom a measurement of MVCq could be of interest before arthroplasty.

## Introduction

Chronic diseases, such as respiratory, cardiovascular, neurological and musculoskeletal diseases (including osteoarthritis) cause a loss in mobility related to a decrease in physical capacity and muscle function [1]. A reduced lean mass or sarcopenia occur in many chronic diseases which induce a further burden on quality of life and morbidity [2]. Detecting a reduced muscle strength is thus an important proxy of health status in subjects with chronic disability.

Osteoarthritis (OA) affects 344 millions of people worldwide, with an incident case rate of 12.5% [3, 4]. It accounts for 19 years of life with disability (DALY) with a peak age range 60–64 years old. OA is associated with a reduced quality of life and impairs daily physical activities in the most disabled patients. Deambulation and balance are more specifically determined by lower limb strength, measured by isometric maximal voluntary contraction of the quadriceps (MVCq). Although well standardized, it requires a specific equipment (i.e. strain gauge), is time-consuming and could be biased by a poor participation. These practical considerations limit MVCq assessment.

Compared to controls, isometric quadriceps force was found decreased of 22–26% [5] and up to 42% [6] in patients with knee osteoarthritis (KOA). Another study concluded to a lower leg extension force with a moderate effect size [7]. Physical tests such as 6 min walk distance, timed up and go, 30s chair to stand test were also altered with a moderate effect size [6]. In hip osteoarthritis (HOA) patients, among several strength deficits, Loureiro et al. [8] showed also a decreased leg extension force compared to controls (effect size 0.82).

A meta-analysis showed that a reduced extensor force was associated with the development of symptomatic KOA [9]. A low MVCq force is also associated with an increased risk of pain and decline in knee function using the specific domains of Western Ontario and McMaster Universities Osteoarthritis Index (WOMAC), during the follow up of these patients [10]. Moreover, maintaining quadriceps function is an outstanding objective in order to improve post-operative outcomes [11]. Additionally, the quadriceps strength of the non-involved limb influenced the recovery of physical function (TUG, stair climbing test) and Knee Injury and Osteoarthritis Outcome Score (KOOS) up to 2 years after knee arthroplasty [12].

The objective of this study was then to find out the determinants of a reduced MVCq in patients before knee or hip surgery for osteoarthritis causing an impairment in daily mobility. A prediction equation of a reduced MVCq allows to establish a score which could be used to screen for those in whom it deserves to be actually measured.

## Methods

Among the patients included in the EVALMOB cohort, we selected those with HOA or KOA at a surgical stage. The EVALMOB study is a 4 years observational cohort study seeking to assess the determinants of mobility loss in chronic diseases (CPP SUD-EST II 2019-A01017-50, Clinical trials NCT04375280). The out-patients in our university hospital were screened through 3 simple questions during medical visit: 1) Can you get up from the squatting position without hands and without any difficulty? 2) Are you limited in activities of daily living? 3) Are you limited in the practice of physical activity? Whether they respond “yes” to one of these questions, the subjects were proposed to participate in a comprehensive assessment of exercise capacity, nutritional status and quality of life and an annual follow-up.The patients gave their written consent to participate in the EVALMOB cohort, and the procedure followed were in accordance with the Helsinki declaration of 1975, as revised in 2000. The including period ranged between August 26th 2020 and May 26th 2023. The subjects were assessed in one setting (Clermont Ferrand Tertiary hospital) by the same investigators (QF, AV, VR, FC).

The inclusion criteria for the present study were then:

- Unilateral HOA or KOA at a surgical stage.
- Reduced mobility as assessed by the positive response at one or more of the screening questions (see above).
- Inclusion in the Evalmob cohort
- Ability to perform the physical tests

The non inclusion criteria were the physical or psychological inability to perform the tests or to respond to questionnaires (french language barrier).

- For each patient, the course of OA pathology was assessed by imaging, classified according to the KELLGREN and LAWRENCE stages [13]. The impact of the disease on impairment of functional abilities was assessed through the Western Ontario McMaster University Osteoarthritis index [WOMAC] [14] in the 2 populations, and the specific questionnaires Knee injury and Osteoarthritis Outcome Score [KOOS] [15] and Hip disability and Osteoarthritis Outcome Score [HOOS] [16] were completed by subjects with KOA and HOA, respectively.

Body Mass Index (BMI, kg.m^-2^) was calculated from measurements of height and weight. Body composition was measured by multifrequency bio-impedance measurement (QuadScan 4000, Bodystat®) [17], allowing to determine muscle mass (kg).

Physical activity level was measured using the Global Physical Activity Questionnaire [GPAQ] [18]. Impairment in quality of life is assessed from the Medical Outcomes Study Short Form questionnaire [SF-36] [19] and mood from the Hospital Anxiety and Depression Scale [HAD] [20].

All participants performed the following functional tests according to established procedures, in the same order: Five Time Sit-to-Stand Test [FTSST] [21], Timed Up and Go test [TUG] [22], maximal gait speed [GS] on the 10-meter walk test [10mWT], and 6-minute walk test [6minWT]. The choice of these field physical tests was justified in 2 reviews on osteoarthritis assessment. This is detailed in Table 1, as well as the references for normal values of every test.

**Table 1:** Justifications of use of physical tests in osteoarthritis and normal value.

	Justification to use	Normal value (reference)
Five Times Sit To Stand Test (FTSST)	++ [29]	Bergland et al. [32]
Timed-Up-and-GO (TUG)	+++ [29] +++[30]	Bohannon R. [33]
Max gait speed (10 meters walk)	++ [29]	Bohannon et al. [34]
6 minute walk test (6mWT)	+++[30] +++[29]	Enright [35]

+++ core set of assessment

++ alternative test in the functional assessment of osteoarthritis according to the reviews by Dobson et al. [30] and Reynaud et al. [29]

Muscle strength was assessed on upper limb (handgrip, Jamar dynamometer) and lower limb (isometric knee extension at 45°, HUMA®). The best peak force among the 3 isometric contractions was retained as the maximum isometric voluntary force (MVCq) expressed in absolute value (Newtons. meter) and as a percentage of predicted value [23]. A reduced MVCq was judged as a measurement lower than one standard deviation of mean value [23] whether it corresponds to the involved or the non-involved limb.

### Statistics

Patient characteristics were expressed as mean and standard-deviation or median and interquartile range for continuous data. The assumption of normality was assessed by using the Shapiro-Wilk test. The continuous variables were compared between independent groups by Student t-test or Mann-Whitney test if the assumptions to apply t-test were not met.

The homoscedasticity was analyzed using the Fisher-Snedecor test. The results were expressed by effect-sizes and 95% confidence intervals. For categorical data, the comparisons were carried out using the chi-squared or Fisher’s exact test. Correlation coefficients (Pearson or Spearman according to statistical distribution) were calculated to evaluate the relationships between physical tests (FTSST, TUG, 6minWT and 10m-GS).

**Fig 1:**
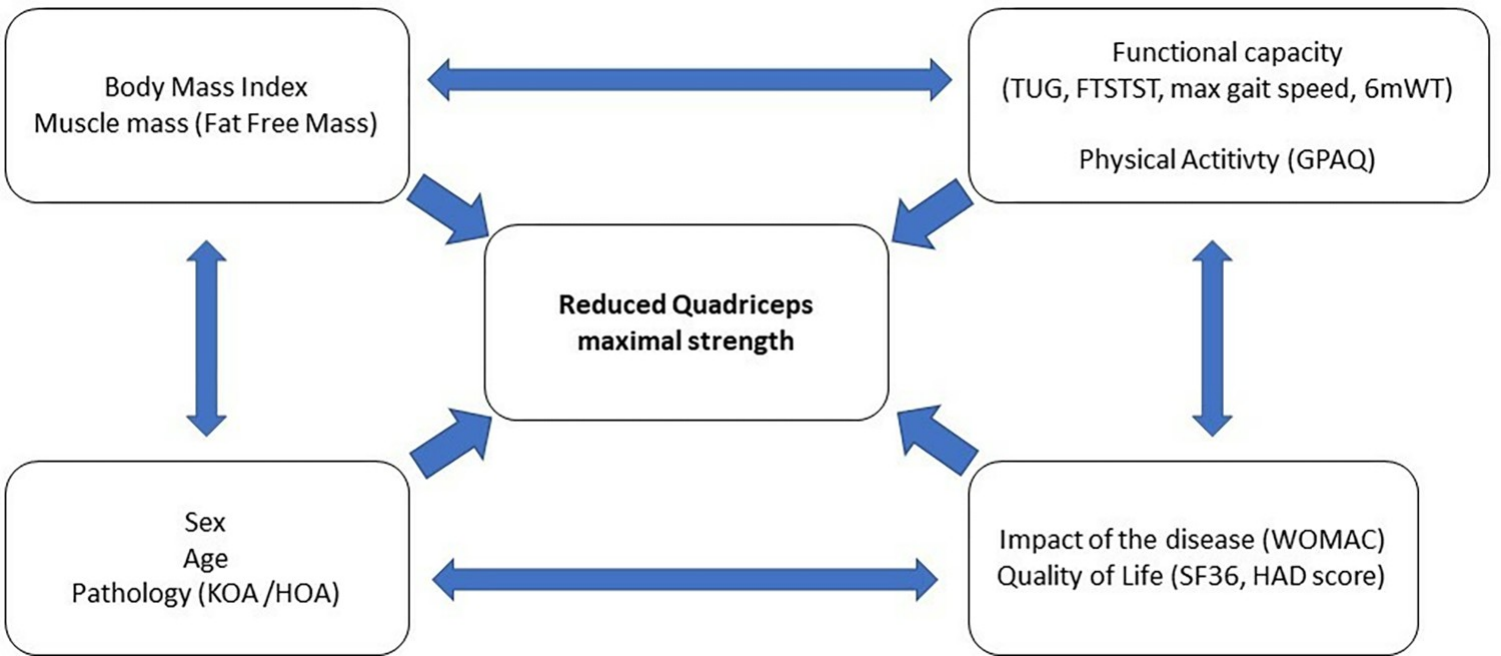
Conceptual framework of predictors of a reduced quadriceps force.

In order to determine factors associated to reduced MVCq force, a generalized linear model (i.e. logistic regression) was carried out on covariates chosen according to univariate results and to their clinically relevance. A particular attention has been paid to the study of multicollinearity and interactions between covariates 1) studying the relationships between the covariables and 2) evaluating the impact to add or delete variables on multivariable model. Results were expressed as odds- ratios and 95% confidence intervals (95%CI) and forest plots were employed to present the results. Sensitivity analyses were performed. A conceptual framework for predictors and the outcome (reduced MVCq) is given in Fig 1.

All analyses were performed using Stata software (Version 13, StataCorp, College Station, TX) for a two-sided Type I error at 5%.

## Results

Among the 538 patients enrolled in the EVALMOB cohort during the study period, 376 subjects suffering of osteoarthritis were included in the present study. The cohort included participants with KOA (n = 247, 66%) or HOA (n = 129, 34%).

Anthropometric data are summarized in Table 2. The mean age was 67±8 years, mean BMI 31.4±6.9 kg/m^2^ and mean muscle mass 51.8±12.4 kg; 53% of patients were considered as obese. According to radiographic stages, 206 (55%) were in stage 3 and 97 (26%) in stage 4 of Kellgren and Lawrence classification.

**Table 2:** Anthropometric data according to the value of quadriceps force.

	Normal QFMV	Reduced QFMV	p	SMD [CI95%]
N (%)	159 (42)	217 (58)		
Pathology HOA	71 (44.7)	58 (26.7)	<0.001	0.38 [0.17, 0.58]
Sex Male	44 (27.7)	110 (50.7)	<0.001	0.48 [0.27, 0.68]
Age (years)	68 [64, 74]	68 [62, 73]	0.319	0.09 [0.11, 0.29]
Height (cm)	161 [157, 168]	165 [159, 172]	0.003	0.30 [0.09, 0.50]
Weight (kg)	75 [64, 84]	89 [81, 101]	<0.001	1.03 [0.79, 1.23]
BMI kg.m^-2^	27.9 [24.5, 30.9]	32.4 [28.9, 38.2]	<0.001	0.96 [0.72, 1.15]
Fat free mass (kg)	44.50 [37.95, 54.35]	54.50 [46.58, 63.65]	<0.001	0.76 [0.54, 0.97]
TUG (s)	8.40 [6.86, 9.61]	10.00 [8.20, 12.15]	<0.001	0.48 [[0.26, 0.67]
FTSST (s)	9.50 [8.15, 12.06]	11.71 [9.50, 15.00]	<0.001	0.49 [0.25, 0.67]
6minWT (m)	466 [413, 539]	406 [316 ; 487]	<0.001	0.60 [0.38, 0.80]
Maximal gait speed (m.s^-1^)	1.92 [1.65 ; 2.25]	1.69 [1.27 ; 2.02]	<0.001	0.51 [0.29, 0.70]
SF 36—PCS	40.4[28.6 ; 53.0]	34.4 [23.8 ; 51.5]	0.02	0.21 [0.01, 0.42]
SF 36—MCS	61.35 [41.25, 77.08]	57.70 [36.87, 78.12]	0.21	0.13 [-0.06, 0.34]
HAD Anxiety	7.00 [5.00, 9.00]	7.00 [5.00, 10.00]	0.75	0.03 [-0.17, 0.23]
HAD Depression	4.00 [2.00, 7.00]	5.00 [3.00, 8.00]	0.01	0.26 [0.06, 0.47]
GPAQ (MET-min/week)	600 [240, 1320]	480 [0, 980]	0.04	- 0.0.1 [-0.23, 0.19]
WOMAC %	48.44 [34.38, 58.33]	44.27 [34.38, 58.33]	0.07	0.21 [0.00, 0.42]

HOA: hip osteoarthritis; TUG: Timed up and Go test; FTSST: five time sit-to-stand test; 6minWT: 6 minute walk distance; maximal GS: maximal gait speed during a 10 meter walk test; SF-36 PCS Physical Component Score. SF-36 MCS Mental Component Score. HAD Hospital Anxiety and Depression Scale; GPAQ Global Physical Activity Questionnaire. WOMAC Western Ontario McMaster University Osteoarthritis index.

For the FTSST, 35 participants were unable to perform the test and were arbitrarily assigned a value of 60 seconds, based on the calculation of the SPPB score. The mean value obtained for FTSST, TUG, maximal gait speed, and 6minWT were 16.12±14.66 s, 12.89±4.69 s, 1.78±0.54 m.s^-1^, and 422±121 meters (92.3±23.5% predicted), respectively. MVCq was measured at 92.3±36.8 N.m, which corresponded to 75.5±25.0% of the expected value.

On the whole population, 159 (42%) had a quadriceps isometric strength considered as normal and 217 (58%) had a decreased strength (Table 2). In the group with altered MVCq, patients presented with a significant higher height, weight, BMI, lean mass, a higher sex ratio (more men), and a higher prevalence in knee compared to hip osteoarthritis (Table 2). Field physical tests were also more altered in this group.

Comparing knee and hip osteoarthritis we found that only BMI (p = 0.03) and WOMAC score (p = 0.02) were significantly different in male (S1 Table).

**Fig 2:**
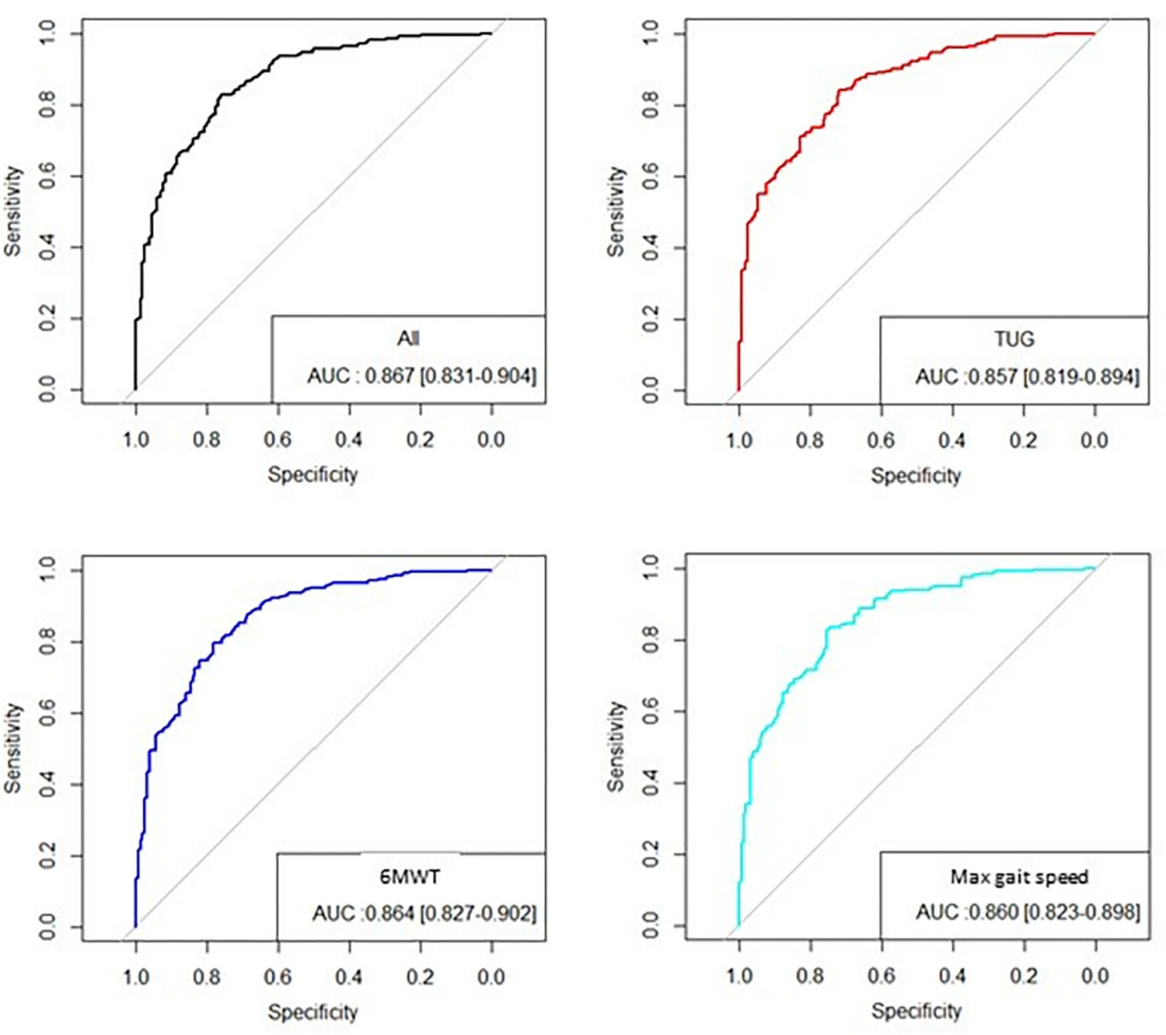
ROC analysis of the predictive model including all physical tests (uppel left) or Five time sit-to-stand test (FTSST) added to Timed-Up-Go test (TUG, upper right), 6 minute walk test (6MWT, lower left) or maximal gait speed (lower right). Similar AUC were obtained with either a model including all or 1 of these tests.

The best logistic regression model taking into account all anthropometric data and functional test results obtained for the entire population for a decreased quadriceps force included age, sex, BMI, FSTSST, TUG, 6minWD and maximal GS. The AUC was 0.87 [95%CI 0.83–0.90] (Fig 2, upper left panel) We found no statistical interaction between the pathology and the parameters included in the regression model, so the same predictive model could be used whatever the joint involved. Interestingly the inclusion of only one physical test (TUG, 6minWT or maximal GS) with FTSTT lead to the same predictive value with an AUC of 0.86 (Fig 2). This means that one of these 3 field tests can be used indifferently to predict a decreased MVCq.

**Fig 3:**
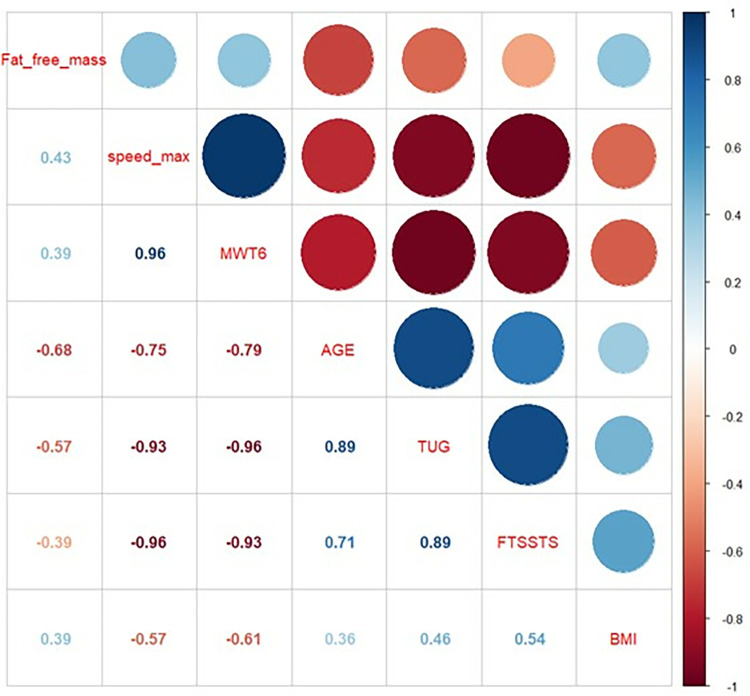
Correlation matrix between parameters included in the predictive model. Speed_max: maximal gait speed; 6minWT = 6-minute walk test; TUG: Timed Up and Go; FTSST: Five time sit-to-stand test; BMI: body mass index.

The correlation matrix between the parameters included in the predictive model is given in Fig 3. Consistently, we found strong significant correlations between the different physical tests (FTSST, TUG, 6minWT and 10m-GS) with regression value exceeding 0.9 (Fig 3).

In the final model we choose maximal gait speed since it can easily be carried out in different settings (AUC 0.86 [0.82–0.90]) (Table 3). According to this model, 79.4% of the subjects were correctly classified; sensitivity and specificity were 83.5% and 74.2%, respectively Thus, the predictive equation for the probability of a reduced MVCq is as follows:

Y = 1/1+ exp[-(-0.051*age -1.25*max gait speed + 0.09*FTSST + 0.16*BMI + 1.1 (for KOA) + 2.41 (for male) -1.79].

As an example, for a man 69 years old man suffering of KOA, with a BMI of 28 kg/m^2^, with a max gait speed of 1.1 m.s^-1^ and a FTSST of 15.2 s the probability of a reduced quadriceps force would be of 94%. On the opposite, for a 70 years old woman suffering of HOA, with a BMI of 30 kg/m^2^, and the same results for max GS and FTSST the probability of a reduced quadriceps force would be of 38%.

**Table 3:** Logistic regression model in the whole population.

	[95% CI]	p-value
Age	-0.05 [-0.09 ; -0.02]	0.004
Sex (male)	2.41 [1.73; 3.08]	<0.001
Disease (KOA)	1.1 [0.51;1.69]	<0.001
BMI	0.16 [0.11 ;0 ;22]	<0.001
Max gait speed	-1.25 [-1.98 ; -0.51]	<0.001
FTSST	0.09 [0.02 ; 0.15	0.006

## Discussion

In patients with hip or knee osteoarthritis, a reduced quadriceps force is associated with poor outcomes after arthroplasty, which argues for its measurement. In the present study, we found a high prevalence of a reduced MVCq associated with a lower physical capacity. We developed a predictive equation including field physical test fora reduced quadriceps strength in patients with osteoarthritis.

We found that 58% of our cohort of patients presented with a decreased of MVCq as defined by a measured below 1 SD of normal values [23]. Previous studies in osteoarthritis compared the involved versus the preserved limb, and an asymmetry of 24% was found [24]. However 2 years after total knee arthroplasty the decline of quadriceps strength of the non-operated limb was larger than that of the operated one and that observed in healthy controls [25]. Osteoarthritis could thus involve both limbs, but to a variable severity at the time of evaluation, and an altered quadriceps force could be due to comorbidities (body composition, sedentary behavior) and not only the consequence of OA. As a consequence, we selected a stricter criterion based on published normal values. As a matter of fact, we found a similar percentage of affected subjects when taking into account a between-limb asymmetry. So we feel confident that our criteria of a MVCq alteration was relevant and probably reflects a severe stage of osteoarthritis in our presurgical cohort of patients. The comparison of our results (prevalence or importance of a decreased MVCq) with previous studies is difficult due to different expression of the measurements (i.e., units or relative to BMI). However, the anthropometry characteristics and physical impairment of our subjects appear similar to others studies, suggesting a similar MVCq as respect of the predictive factors of quadriceps strength.

Comparing the site of OA, we found a larger number of patients with a decreased QMVC in knee than in hip arthrosis (supplemental file 1). In a systematic review, Loureiro et al concluded of a generalized muscle weakness of the affected leg in patients with HOA, but in mild to moderate severity of hip arthrosis no between leg asymmetry [8, 26]. So, our findings of a lower strength of knee extensors in HOA appear consistent. Since, the determinants of quadriceps force were similar in HOA and in KOA, both pathologies were gathered to increase the significance of our results in lower limb osteoarthrosis whatever the joint affected.

Among the factors associated with a decreased QMVC, BMI had the higher odd ratio (1.18) after the age of the subject. Obesity is a common comorbidity in OA and affected 53% of our population. As a consequence, a higher lean mass was also present in those with a higher BMI and the muscle mass (muscle mass index) was not significant in predicting MVCq. Lower limb muscle strength and volume appear altered to a similar level in OA (compared to non-affected limb or healthy control) [8] which explain why BMI was a stronger predictor of quadriceps force than whole body muscle mass.

Besides anthropometric data, we found that physical capacity predicts MVCq and one can substitute FSTSS or 6MWD by TUG or maximal gait speed and obtain the same prediction (similar AUC). In a perspective of a screening test in a low equipment setting one can use simple field test to detect a decreased QMVC in patients with OA and refer for an actual measurement only those with an altered physical performance. This strategy should help in a larger screening of quadriceps alteration in presurgical stage of OA in order to improve muscle function before the surgical procedure or to postpone the intervention by improving the function and the quality of life and reducing pain [27].

Dobson et al. [28] found that self-paced walk test (40 m), maximal gait speed, 30 s-chair stand test, timed up and go test 12-step stair test were validated to evaluate patients with OA. Recently, our team reviewed the physical tests to assess patients before and after knee arthroplasty and we concluded that 6-min walk test and TUG were the most validated tests [29]. Thus, the current results extended this review and it appears that whatever the physical field test, an altered performance predicts a reduced quadriceps strength in these patients. This allows to spread the evaluation in various local facilities (space, equipment…) with a similar predictive value.

Based on the Osteoarthritis Research Society International (OARSI) recommendations [30] and the known impact on surgical recovery after arthroplasty, our results argue for the search of a reduced quadriceps force through field physical test and the proposed predictive equation in order to evaluate the need to initiate a presurgical physiotherapy.

### Strengths and limits of the study

The strength of our study is a comprehensive assessment of the patients including physical capacity, quality of life, disease severity score, body composition which allows to include a large panel of parameters in the predictive model. The strong correlations between the chosen physical tests explained that the physical tests could be changed in the statistical model without a loss of precision in the prediction of a reduced quadriceps force.

We acknowledge several weaknesses in our study. Firstly, it is a monocentric study limiting the generalization of the results. However, this allows a strict methodology which lowers the risk of measurement errors. Secondly, the population studied could be biased by the severity of OA, at a surgical stage, and thus a low level of daily life physical activity. This would further reduce quadriceps strength. Due to different expression of results, we could not compare MVCq with that measured in previous studies. However, the clinical impact of a reduced MVCq appears mainly at this stage of OA when MVCq proved to be a prognostic outcome. Lastly, muscle mass was determined by bio-impedance analysis which has a lower precision than dual X ray absorptiometry. In the range of BMI of our patients, BIA overestimated fat free mass on average of 7 kg [31]. Whether this bias of measurement explains the lack of relation between FFM and MVCq remains to be established.

## Conclusion

In patients with osteoarthritis at a presurgical stage, a reduced quadriceps strength is highly prevalent and is determined by age, sex, BMI, five sit to stand test and maximal gait speed. Timed up and go test or 6 minutes walk distance could be alternatively included in the regression model with the same precision of prediction. We proposed a predictive equation for calculating the probability of a reduced MVCq in order to screen for the patients in whom an effective measurement of MVCq could be proposed before arthroplasty and/or physiotherapy could be initiate.

## Supporting information

S1 Table Characteristics, physical capacity tests in patients with knee osteoarthrosis and hip osteoarthrosis. ^$^ significantly different between knee and hip osteoarthritis, TUG: Timed up and Go test; FTSST: five time sit-to-stand test; 6minWT: 6 minute walk distance; maximal GS: maximal gait speed during a 10 meter walk test; MVCq: maximal voluntary contraction force of the quadriceps; SF-36 PCS Physical Component Score. SF-36 MCS Mental Component Score. HAD Hospital Anxiety and Depression Scale; GPAQ Global Physical Activity Questionnaire. HOOS: Hip Injury and Osteoarthritis Outcome Score; KOOS: Knee Injury and Osteoarthritis Outcome Score.
(PDF)

## References

[1] Grimmer M, Riener R, Walsh CJ, Seyfarth A. Mobility related physical and functional losses due to aging and disease—a motivation for lower limb exoskeletons. J Neuro Engineering Rehabil 2019;16:2. doi: 10.1186/s12984-018-0458-8 30606194 PMC6318939

[2] Gallo-Villegas JA, Calderón JC. Epidemiological, mechanistic, and practical bases for assessment of cardiorespiratory fitness and muscle status in adults in healthcare settings. Eur J Appl Physiol 2023;123:945–64. doi: 10.1007/s00421-022-05114-y 36683091 PMC10119074

[3] Cieza A, Causey K, Kamenov K, Hanson SW, Chatterji S, Vos T. Global estimates of the need for rehabilitation based on the Global Burden of Disease study 2019: a systematic analysis for the Global Burden of Disease Study 2019. Lancet Lond Engl 2020;396:2006–17. doi: 10.1016/S0140-6736(20)32340-0 33275908 PMC7811204

[4] Liu S, Wang B, Fan S, Wang Y, Zhan Y, Ye D. Global burden of musculoskeletal disorders and attributable factors in 204 countries and territories: a secondary analysis of the Global Burden of Disease 2019 study. BMJ Open 2022;12:e062183. doi: 10.1136/bmjopen-2022-062183 35768100 PMC9244680

[5] Alnahdi AH, Zeni JA, Snyder-Mackler L. Muscle Impairments in Patients With Knee Osteoarthritis. Sports Health 2012;4:284–92. doi: 10.1177/1941738112445726 23016099 PMC3435919

[6] Mau-Moeller A, Jacksteit R, Jackszis M, Feldhege F, Weippert M, Mittelmeier W, et al. Neuromuscular function of the quadriceps muscle during isometric maximal, submaximal and submaximal fatiguing voluntary contractions in knee osteoarthrosis patients. PloS One 2017;12:e0176976. doi: 10.1371/journal.pone.0176976 28505208 PMC5432168

[7] Vårbakken K, Lorås H, Nilsson KG, Engdal M, Stensdotter AK. Relative difference among 27 functional measures in patients with knee osteoarthritis: an exploratory cross-sectional case-control study. BMC Musculoskelet Disord 2019;20:462. doi: 10.1186/s12891-019-2845-0 31638971 PMC6805424

[8] Loureiro A, Constantinou M, Diamond LE, Beck B, Barrett R. Individuals with mild-to-moderate hip osteoarthritis have lower limb muscle strength and volume deficits. BMC Musculoskelet Disord 2018;19:303. doi: 10.1186/s12891-018-2230-4 30131064 PMC6103991

[9] Øiestad BE, Juhl CB, Eitzen I, Thorlund JB. Knee extensor muscle weakness is a risk factor for development of knee osteoarthritis. A systematic review and meta-analysis. Osteoarthritis Cartilage 2015;23:171–7. doi: 10.1016/j.joca.2014.10.008 25450853

[10] Culvenor AG, Ruhdorfer A, Juhl C, Eckstein F, Øiestad BE. Knee Extensor Strength and Risk of Structural, Symptomatic, and Functional Decline in Knee Osteoarthritis: A Systematic Review and Meta-Analysis. Arthritis Care Res 2017;69:649–58. doi: 10.1002/acr.23005 27563843

[11] Paravlic AH, Meulenberg CJ, Drole K. The Time Course of Quadriceps Strength Recovery After Total Knee Arthroplasty Is Influenced by Body Mass Index, Sex, and Age of Patients: Systematic Review and Meta-Analysis. Front Med 2022;9:865412. doi: 10.3389/fmed.2022.865412 35692543 PMC9174520

[12] Zeni JA Jr, Snyder-Mackler L. Early Postoperative Measures Predict 1- and 2-Year Outcomes After Unilateral Total Knee Arthroplasty: Importance of Contralateral Limb Strength. Phys Ther 2010;90:43–54. doi: 10.2522/ptj.20090089 19959653 PMC2802824

[13] Kellgren JH, Lawrence JS. Radiological Assessment of Osteo-Arthrosis. Ann Rheum Dis 1957;16:494–502. doi: 10.1136/ard.16.4.494 13498604 PMC1006995

[14] Bellamy N, Buchanan WW, Goldsmith CH, Campbell J, Stitt LW. Validation study of WOMAC: a health status instrument for measuring clinically important patient relevant outcomes to antirheumatic drug therapy in patients with osteoarthritis of the hip or knee. J Rheumatol 1988;15:1833–40. 3068365

[15] Collins NJ, Prinsen C a. C, Christensen R, Bartels EM, Terwee CB, Roos EM. Knee Injury and Osteoarthritis Outcome Score (KOOS): systematic review and meta-analysis of measurement properties. Osteoarthritis Cartilage 2016;24:1317–29. doi: 10.1016/j.joca.2016.03.010 27012756

[16] Nilsdotter AK, Lohmander LS, Klässbo M, Roos EM. Hip disability and osteoarthritis outcome score (HOOS)—validity and responsiveness in total hip replacement. BMC Musculoskelet Disord 2003;4:10. doi: 10.1186/1471-2474-4-10 12777182 PMC161815

[17] Janssen I, Heymsfield SB, Baumgartner RN, Ross R. Estimation of skeletal muscle mass by bioelectrical impedance analysis. J Appl Physiol Bethesda Md 1985 2000;89:465–71. doi: 10.1152/jappl.2000.89.2.465 10926627

[18] Bull FC, Maslin TS, Armstrong T. Global physical activity questionnaire (GPAQ): nine country reliability and validity study. J Phys Act Health 2009;6:790–804. doi: 10.1123/jpah.6.6.790 20101923

[19] Perneger TV, Leplège A, Etter JF, Rougemont A. Validation of a French-language version of the MOS 36-Item Short Form Health Survey (SF-36) in young healthy adults. J Clin Epidemiol 1995;48:1051–60. doi: 10.1016/0895-4356(94)00227-h 7775992

[20] Zigmond AS, Snaith RP. The hospital anxiety and depression scale. Acta Psychiatr Scand 1983;67:361–70. doi: 10.1111/j.1600-0447.1983.tb09716.x 6880820

[21] Whitney SL, Wrisley DM, Marchetti GF, Gee MA, Redfern MS, Furman JM. Clinical measurement of sit-to-stand performance in people with balance disorders: validity of data for the Five-Times-Sit-to-Stand Test. Phys Ther 2005;85:1034–45. 16180952

[22] Podsiadlo D, Richardson S. The timed “Up & Go”: a test of basic functional mobility for frail elderly persons. J Am Geriatr Soc 1991;39:142–8. 1991946 10.1111/j.1532-5415.1991.tb01616.x

[23] Hogrel J-Y, Payan CA, Ollivier G, Tanant V, Attarian S, Couillandre A, et al. Development of a French isometric strength normative database for adults using quantitative muscle testing. Arch Phys Med Rehabil 2007;88:1289–97. doi: 10.1016/j.apmr.2007.07.011 17908571

[24] Maffiuletti NA, Bizzini M, Widler K, Munzinger U. Asymmetry in Quadriceps Rate of Force Development as a Functional Outcome Measure in TKA. Clin Orthop Relat Res 2010;468:191. doi: 10.1007/s11999-009-0978-4 19597897 PMC2795845

[25] Farquhar S, Snyder-Mackler L. The Chitranjan Ranawat Award: The Nonoperated Knee Predicts Function 3 Years after Unilateral Total Knee Arthroplasty. Clin Orthop Relat Res 2010;468:37. doi: 10.1007/s11999-009-0892-9 19472024 PMC2795832

[26] Loureiro A, Mills PM, Barrett RS. Muscle weakness in hip osteoarthritis: a systematic review. Arthritis Care Res 2013;65:340–52. doi: 10.1002/acr.21806 22833493

[27] Hurley M, Dickson K, Hallett R, Grant R, Hauari H, Walsh N, et al. Exercise interventions and patient beliefs for people with hip, knee or hip and knee osteoarthritis: a mixed methods review. Cochrane Database Syst Rev 2018. doi: 10.1002/14651858.CD010842.pub2 29664187 PMC6494515

[28] Dobson F, Hinman RS, Hall M, Terwee CB, Roos EM, Bennell KL. Measurement properties of performance-based measures to assess physical function in hip and knee osteoarthritis: a systematic review. Osteoarthritis Cartilage 2012;20:1548–62. doi: 10.1016/j.joca.2012.08.015 22944525

[29] Reynaud V, Verdilos A, Pereira B, Boisgard S, Costes F, Coudeyre E. Core Outcome Measurement Instruments for Clinical Trials of Total Knee Arthroplasty: A Systematic Review. J Clin Med 2020;9:2439. doi: 10.3390/jcm9082439 32751523 PMC7463550

[30] Dobson F, Hinman RS, Roos EM, Abbott JH, Stratford P, Davis AM, et al. OARSI recommended performance-based tests to assess physical function in people diagnosed with hip or knee osteoarthritis. Osteoarthritis Cartilage 2013;21:1042–52. doi: 10.1016/j.joca.2013.05.002 23680877

[31] Achamrah N, Colange G, Delay J, Rimbert A, Folope V, Petit A, et al. Comparison of body composition assessment by DXA and BIA according to the body mass index: A retrospective study on 3655 measures. PLOS ONE 2018;13:e0200465. doi: 10.1371/journal.pone.0200465 30001381 PMC6042744

[32] Bergland A, Strand BH. Norwegian reference values for the Short Physical Performance Battery (SPPB): the Tromsø Study. BMC Geriatr 2019;19:216. 10.1186/s12877-019-1234-8. 31395008 PMC6686475

[33] Bohannon RW. Reference Values for the Timed Up and Go Test: A Descriptive Meta-Analysis. J Geriatr Phys Ther 2006;29:64–8. doi: 10.1519/00139143-200608000-00004 16914068

[34] Bohannon RW, Andrews AW, Thomas MW. Walking speed: reference values and correlates for older adults. J Orthop Sports Phys Ther 1996;24:86–90. doi: 10.2519/jospt.1996.24.2.86 8832471

[35] Enright PL, Sherrill DL. Reference equations for the six-minute walk in healthy adults. Am J Respir Crit Care Med 1998;158:1384–7. doi: 10.1164/ajrccm.158.5.9710086 9817683

