# Supplementary material for: Abnormality in field physical test predicts a reduced quadriceps strength in patients with hip- or knee-osteoarthritis. A prospective observational study
Source: PLoS One. 2024 Dec 27;19(12):e0314524. doi: 10.1371/journal.pone.0314524 (PMC11676513; doi:10.1371/journal.pone.0314524)
Supplement: S1 Table — $ significantly different between knee and hip osteoarthritis, TUG: Timed up and Go test; FTSST: five time sit-to-stand test; 6minWT: 6 minute walk distance; maximal GS: maximal gait speed during a 10 meter walk test; MVCq: maximal voluntary contraction force of the quadriceps; SF-36 PCS Physical Component Score. SF-36 MCS Mental Component Score. HAD Hospital Anxiety and Depression Scale; GPAQ Global Physical Activity Questionnaire. HOOS: Hip Injury and Osteoarthritis Outcome Score; KOOS: Knee Injury and Osteoarthritis Outcome Score. (PDF) [file pone.0314524.s001.pdf]

	KOA		HOA	
	Males	Females	Males	Females
Age (years)	68 [53 – 83]	67 [34 – 88]	67 [45 – 79]	66 [33– 80]
BMI (kg/m2)	31.18 (5.25)	32.55 (7.93)	29.45 (4.70)[$]	30.83 (7.43)
Fat free mass (kg)	64.2 (9.0)	44.3 (7.3)	62.1 (8.6)	43.3 (7.0)
TUG (s)	9.2 ±3.32	10.7 ±5.3	8.9 ±2.6	11.4 ±5.7
FTSST (s)	10.7±3.1	12.3±5.0	10.8±4.8	12.8±5.7
6minWT (meter)	461.3±105.1	393.4±113.9	472.2±112.3	390.0±134.4
Maximal GS ($m.s^{-1}$)	1.21±0.2	1.06±0.3	1.19±0.3	1.09±0.3
MVCq right (N.m)	113.0±32.5	71.8±24.6	127.9±36.4	79.8±26.9
MVCq left (N.m)	115.7±34.6	71.6±22.9	123.5±38.6	77.7±25.5
SF 36 - PCS	47.16 (18.17)	38.44 (17.62)	42.92 (18.63	37.42 (16.74)
SF 36 - MCS	60.34 (21.46)	51.92 (22.63)	55.60 (20.65)	49.44 (21.53)
HAD Anxiety	5.93 (3.46)	8.00 (3.90)	6.74 (3.01)	7.67 (3.44)
HAD Depression	4.77 (3.21)	5.53 (3.99)	4.81 (2.94)	5.63 (3.98)
GPAQ (MET-min/week)	1334 (1932)	931 (1431)	1252 (2560)	726 (1016)
HOOS %			40.25 (16.69)	35.90 (13.40)
KOOS %	40.25 (16.69)	35.90 (13.40)		
